# The effect of meditation on exam anxiety of nursing students: a randomized clinical trial

**DOI:** 10.1007/s11845-026-04308-z

**Published:** 2026-03-24

**Authors:** Pouran Varvani Farahani, Candan Ozturk

**Affiliations:** 1 Department of Nursing, Cyprus International University, Nicosia, 99258 North Cyprus; 2 Faculty of Nursing, Near East University, Lefkoşa/KKTC, Mersin 10, Türkiye

**Keywords:** Meditation, Exam Anxiety, Nursing Students, Complementary Therapies, Mental Health

## Abstract

**Background:**

Nursing students frequently encounter significant levels of test anxiety, which can impact their academic success and overall well-being. Twin Heart Meditation is a technique for relaxation that might assist in alleviating anxiety.

**Aims:**

This research aim to assess the influence of Twin Heart Meditation on exam anxiety in nursing students.

**Methods:**

This double-blind clinical trial was conducted on 100 nursing students. Participants were randomly assigned into two groups: a control group (n = 50) and an experimental group (n = 50). In the experimental group, participants engaged in meditation for 30 minutes, three times per week, over a one-week period. Both groups underwent assessments of exam anxiety before and after the intervention, utilizing the Spielberg State-Trait Anxiety Inventory. Data were analyzed using SPSS version 25, employing paired and independent t-tests, with P < 0.05 considered statistically significant.

**Results:**

There was no significant difference in exam anxiety scores between the two groups before to the intervention (P = 0.473). Following the intervention, the mean exam anxiety score in the experimental group significantly decreased from 56.52± 13.91 to 50.22± 11.54 (P < 0.001), whereas the control group showed no statistically significant change, with scores from 58.48± 13.27 to 58.42± 13.21 (P = 0.371).. Moreover, an independent t-test revealed a statistically significant difference in post-intervention exam anxiety scores between the two groups (P ≤ 0.001).

**Conclusions:**

Meditation can be regarded as an effective, non-invasive approach to alleviate exam anxiety in nursing students, which may lead to improvements in their academic success and general mental health.

## Introduction

Students occupy a unique position in society as the creative and intellectual forces. For this reason, it is essential to safeguard students’ mental well-being while they acquire knowledge and enhance their scientific understanding. One of the factors that can lead to difficulties and exacerbate mental distress, potentially resulting in a decrease in individual performance, is exam anxiety [1, 2]. Exam anxiety is one of the most common stress-related conditions in this population and is characterized by excessive worry, emotional distress, and cognitive interference during evaluative situations [3]. High levels of exam anxiety have been associated with impaired concentration, reduced academic performance, and diminished psychological well-being [4].

Recent studies indicate that exam anxiety is highly prevalent among nursing and medical students worldwide, affecting approximately 25–40% of undergraduate students in health-related disciplines [5]. Meta-analytic evidence further suggests that nearly one-third (33.8%) of individuals reporting clinically relevant anxiety symptoms are medical or nursing students, with the highest prevalence observed in students from the Middle East and Asia [6]. According to numerous studies in the academic sector, exam anxiety has been associated with negative academic and mental health outcomes, particularly in high-demand educational environments. These findings underscore the need for effective, low-risk, and accessible interventions to manage anxiety in student populations [7, 8] .

In recent years, complementary and alternative medicine (CAM) approaches have gained increasing attention as supportive strategies for anxiety management [9, 10]. Based on the results of recent studies, meditation-based interventions may reduce anxiety and stress while being non-invasive, cost-effective, and easy to implement [11]. Meditation is a form of CAM therapy that explores the connection between the mind and body. It is a deliberate mental activity that can induce physiological alterations over time by focusing one’s attention on specific stimuli (auditory and visual) [12]. There are several types; one such type is twin hearts meditation. This practice is founded on the idea that concentrating on and energizing two chakras (the heart and crown) enhances brain functionality, supports the nervous system and thymus gland, boosts bodily vitality, and, in turn, diminishes stress and anxiety [13]. However, few randomized controlled trials have examined the full effects of Twin Hearts meditation on anxiety. As far as we are aware, there are no published statistics on Twin Heart Meditation affects nursing students’ exam anxiety. The present study aimed to evaluate the effect of Twin Hearts Meditation on exam anxiety among nursing students.

It was hypothesized that nursing students who participated in Twin Hearts Meditation would demonstrate significantly lower exam anxiety scores compared with students in the control group.

## Methods

The present study was designed as a double-blind randomized controlled trial. Among 120 eligible undergraduate nursing students, 100 students who met the inclusion criteria were recruited using a convenience sampling method. Written informed consent was obtained from all participants prior to enrollment.

Following recruitment, participants were randomly allocated to either the experimental or control group using a simple randomization procedure. A sequence of numbers from 1 to 100 was generated, and each number was placed on identical cards. The cards were thoroughly mixed in a container to ensure randomness. Numbers were then drawn sequentially and assigned alternately to the experimental and control groups until equal group sizes were achieved (*n* = 50 per group). To minimize allocation bias, group assignments were concealed from the investigator responsible for data collection. Furthermore, the statistician analyzing the data remained blinded to group allocation, and datasets were coded as x1 and x2 during analysis.

**Fig. 1:**
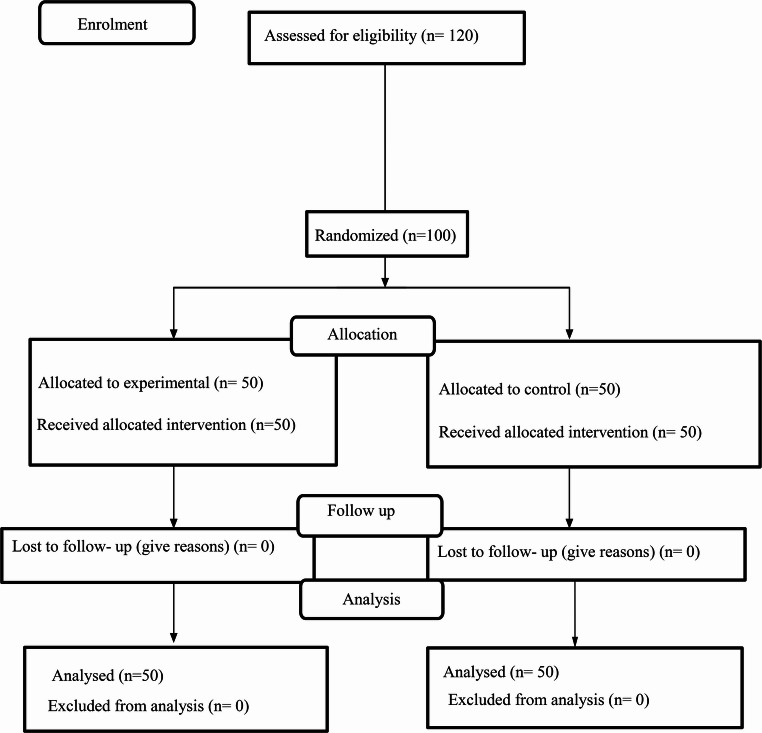
CONSORT flow diagram of the study participants

To determine the sample size based on the findings of a similar study [14] and the mean comparison formula, the first type error level was 0.05, the power of the test was 80%, and with (S1 = 9.61, S2 = 10.4, µ1 = 42.03.5, µ2 = 47.7). The sample size in each group is 48 people, which is 50 people in each group considering 5% dropout (Fig. 1).

$$n=\frac{\left({{z}_{1-\frac{\alpha}{2}}+{z}_{1-\beta})}^{2}\right({{s}_{1}}^{2}+{{s}_{2}}^{2})}{{d}^{2}}$$

### Inclusion criteria

1. All undergraduate nursing students in their seventh semester, regardless of gender, registered in the English-language program at the Department of Nursing, Near East University.
2. Students who are not utilizing any alternative stress alleviation strategies, including progressive muscle relaxation, medication therapy, or comparable approaches, to cope with anxiety related to exams.
3. Participants who willingly chose to take part in the research and gave their informed consent.

### Exclusion criteria

1. Practicing meditation fewer than 3 times each week.
2. Reluctant to maintain collaboration on the project,
3. Utilizing any psychiatric medications (anti-depressants …), psychological well-being.
4. Grieving, particularly the loss of dear ones or family members in the initial stage.

### Ethical considerations

The research received ethical clearance from the Near East University Ethics Review Board with the approval number NUE/2023/114–1736. The initiative closely followed the ethical standards established by the Council of Ethics and was carried out in complete accordance with the principles outlined in the Declaration of Helsinki (1964). Additionally, the study was registered with ClinicalTrials.gov under the ID NCT07098208.

The study’s aim and methodology were explained to the participants. Participants were given the assurance that they could leave the study at any moment without incurring any fees. Finally, Participants who voluntarily stated their involvement in the study provided written informed consent. The study was carried out in accordance with the Declaration of Helsinki’s principles [15] .

### Study tools

1. **The questionnaire for collecting demographic**: This section of the questionnaire gathered demographic details regarding the students, such as age, gender, marital status, and education history.
2. ** The Spielberg State-Trait Anxiety Inventory (STAI)**: the Spielberg State-Trait Anxiety Inventory (STAI) is a widely used self-report instrument designed to assess two distinct dimensions of anxiety: state anxiety and trait anxiety. The inventory consists of 40 items, with 20 items measuring state anxiety defined as a transient emotional condition influenced by situational factors and 20 items measuring trait anxiety, which reflects a relatively stable tendency to experience anxiety.

Each subscale is scored independently. Items are rated on a 4-point Likert scale. For the state anxiety subscale, response options range from 1 (not at all) to 4 (very much so), reflecting how the respondent feels at the moment. For the trait anxiety subscale, responses range from 1 (almost never) to 4 (almost always), indicating how the respondent generally feels. The range is from 40 to 160 points. A score of 40 to 79.9 is considered mild anxiety, 80 to 119.9 is moderate exercise, and 120 to 160 is considered severe.

The STAI is widely used and suitable to administer in clinical practice and research, because of its relatively brief self-report scale to assess both state and trait anxiety. Numerous translations of the STAI have been done, including Greek, Dutch, Japanese, Chinese, Persian, Turkish and Malaysian. These studies consistently found acceptable reliability of the translated versions of the STAI. Thomas & Cassady conducted a project to validate and reliability for English version tool in 2021, obtaining excellent internal consistency and Cronbach’s Alpha in all parts as 0.96 [16].

### Intervention

The participants involved underwent both theoretical and practical instruction in Twin Hearts meditation from the researcher, who possesses expertise in this meditation style. The learners received personalized, in-person training on Twin Hearts meditation. Following this, they engaged in Twin Hearts meditation for thirty minutes with the guidance of the researcher, utilizing an MP3 player. They practiced the meditation at least three times per week (for 30 min each session) during a span of one week. The meditation process was carried out as follows: They performed physical exercises for 5 min.

1. They engaged in praying.
2. They sanctified the earth and filled it with love and warmth while gaining knowledge about the heart chakra (activating the heart chakra).
3. They made the earth holy and full of love and affection while being informed about the crown chakra (activating the crown chakra).
4. They concentrated on Amen.
5. They conveyed appreciation for his kindness.
6. They performed physical exercises for 5 min [13, 17].

The participants in both the experimental and control groups completed the Spielberger State–Trait Anxiety Inventory (STAI) one week and two hours before the first examination. During the intervention period, students in the control group received no meditation or relaxation intervention and continued their routine academic and examination preparation activities as part of the standard university program. In contrast, participants in the experimental group, in addition to the standard university programs, participated in Twin Hearts Meditation sessions.

### Data Analysis

The results attained from each group were analyzed by a person blind to the intervention method. The collected data from the survey were analyzed using Statistical Package for the Social Sciences (SPSS) software version 25.0. The normality of the data was assessed using the Kolmogorov-Smirnov test. After confirming normal distribution, descriptive statistics, Chi-square test, paired and independent t-tests were employed for data analysis. A significance level of *P* < 0.05 was applied to determine statistical significance.

## Results

All 100 nursing students who participated in the study were included into the ultimate analysis. In the experimental group, the average age was 22.9 ± 3.9 years, while in the control group, the average age was 23.1 ± 3.9 years. The independent t-test showed no significant difference in age between the two groups (*P* > 0.05). Regarding gender distribution, 20.5% (12) of the experimental group were male and 79.5% (38) were female, whereas 23.1% (10) of the control group were male and 76.9%(40) were female; these differences were not statistically meaningful (*P* > 0.05). In terms of marital status, most participants were single, with 97.4% (49) in the experimental group and 92.3% (46) in the control group, and no statistically significant difference was observed between the groups (*P* > 0.05). Furthermore, there were no significant differences in educational level between the two groups (*P* > 0.05) (Table 1).

**Table 1:** Comparison of sociodemographic characteristics between experimental and control groups

Variable	Experimental group (*n* = 50) *N* (%)	Control group (*n* = 50) *N* (%)	*P* value
Marital status			0.790*
Single	49 (97.4)	46 (92.3)	
Married	1 (2.6)	3 (7.7)	
Sex			0.079*
Male	12 (20.5)	10 (23.1)	
Female	38 (79.5)	40 (76.9)	
Age (year) (Mean ± SD)	22.9 ± 3.9	23.1 ± 3.9	0.879**

*Chi square; **Independent t-test

The mean exam anxiety score in the experimental group was 56.52 ± 13.91 before meditation and decreased to 50.22 ± 11.54 after meditation, and the difference was significant (*P* < 0.001). The mean exam anxiety score in the control group was 58.48 ± 13.27 before meditation and 58.42 ± 13.21 after meditation, and the difference was no significant (*P* = 0.371). The independent t-test results showed a statistically significant difference in the post-test exam anxiety scores between the two groups (*P* < 0.001) (Table 2).

**Table 2:** Mean anxiety scores for the two study groups before and after the intervention

Variables	Group	Mean ± SD		P value*	t^*^
		Pretest	Post test		
Anxiety scores	Experimental	56.52 ± 13.91	50.22 ± 11.54	*<*0.001	6.968
	Control	58.48 ± 13.27	58.42 ± 13.21	0.371	0.903
	P value**	0.473	*<*0.001		
	t**	0.721	3.305		

*Paired t-test; **Independent t-test

## Discussion

The study shows that there was no significant variation in the demographic features of the two groups. Furthermore, it was determined that there was no statistically meaningful difference in the pre-intervention exam anxiety scores between the two groups.

The study’s findings revealed that both the experimental and control groups experienced exam anxiety before the intervention. However, the intervention group showed a significant reduction in anxiety scores after participating in the meditation sessions. In contrast, only a slight decrease was observed in the control group. These findings indicate that meditation was effective in reducing exam anxiety among nursing students. The observed reduction in anxiety is consistent with previous research demonstrating the psychological benefits of meditation-based interventions. Studies conducted among nursing students practicing Twin Hearts Meditation have reported improvements in relaxation and psychological well-being [18]. Similar findings have been observed in other populations, including breast cancer patients, where meditation was associated with reduced anxiety and improved quality of life [19], and transgender women, where even a single meditation session resulted in decreased anxiety and improved emotional balance [17].

In addition, a study among university students found that Taizé meditation significantly decreased anxiety levels [20]. Similarly, research involving pre-healthcare students practicing guided mindfulness meditation showed reductions in stress and anxiety symptoms [21]. Another investigation, which employed contemplative meditation techniques focused on positive emotional awareness, demonstrated enhanced emotional regulation an essential factor in managing academic anxiety [22]. The findings of the present study align with these previous studies and reinforce the idea that meditation can serve as a beneficial non-pharmacological intervention for stress and anxiety, particularly in academic settings.

Although meditation techniques varied across studies, the present findings contribute to the limited evidence specifically examining Twin Hearts Meditation for exam anxiety among nursing students. This form of meditation emphasizes personal responsibility, self-regulation, and active engagement in managing psychological well-being. By encouraging students to take an active role in stress management, meditation may help support emotional stability, concentration, and mental clarity, which can be beneficial for academic functioning in high pressure educational environments.

### Limitations

Despite these positive outcomes, several limitations should be acknowledged. The short duration of the intervention only one week may not adequately reflect the long-term benefits of meditation. Future studies should involve more diverse populations and include long-term follow-up to evaluate the sustained effects of meditation on exam anxiety and academic performance.

## Conclusions

According to the study’s findings, Twin Hearts Meditation appears to be an effective complementary intervention for reducing exam anxiety among nursing students. The results showed that students who practiced Twin Hearts Meditation experienced a significant decrease in anxiety levels compared to those in the control group, indicating its potential as a supportive strategy for managing academic stress. As a non-invasive, cost-effective, and self-regulated technique, meditation supports the development of inner calm, focus, and emotional resilience key factors for success in high-pressure academic environments. Nursing educators and academic staff can play a crucial role in introducing and guiding students through meditation practices, fostering a more balanced and health-promoting learning environment.

### Recommendations

Future studies should include larger and more diverse samples, longer follow-up periods, and objective measures of anxiety to further examine the effectiveness of meditation-based interventions. Incorporating meditation programs into nursing education may also be explored as a supportive strategy for managing exam anxiety.
